# Identification of glycosylated marker proteins of epithelial polarity in MDCK cells by homology driven proteomics

**DOI:** 10.1186/1471-2091-7-8

**Published:** 2006-03-13

**Authors:** Joachim Füllekrug, Anna Shevchenko, Andrej Shevchenko, Kai Simons

**Affiliations:** 1 Max-Planck-Institute of Molecular Cell Biology and Genetics, Dresden, Germany; 2 University of Heidelberg, Internal Medicine IV, Molecular Cell Biology Group, Im Neuenheimer Feld 345, 69120 Heidelberg, Germany

## Abstract

**Background:**

MDCK cells derived from canine kidney are an important experimental model system for investigating epithelial polarity in mammalian cells. Monoclonal antibodies against apical gp114 and basolateral p58 have served as important tools in these studies. However, the molecular identity of these membrane glycoproteins has not been known.

**Results:**

We have identified the sialoglycoprotein gp114 as a dog homologue of the carcinoembryonic antigen-related cell adhesion molecule (CEACAM) family. Gp114 was enriched from tissue culture cells by subcellular fractionation and immunoaffinity chromatography. The identification was based on tandem mass spectrometry and homology based proteomics. In addition, the p58 basolateral marker glycoprotein was found to be the β subunit of Na^+^K^+^-ATPase.

**Conclusion:**

Gp114 has been characterized previously regarding glycosylation dependent trafficking and lipid raft association. The identification as a member of the canine CEACAM family will enable synergy between the fields of epithelial cell biology and other research areas. Our approach exemplifies how membrane proteins can be identified from species with unsequenced genomes by homology based proteomics. This approach is applicable to any model system.

## Background

Madin-Darby canine kidney (MDCK) cells are the best established mammalian model for studying epithelial cell biology. MDCK cells differentiate into polarized cells within a few days when grown on semi-permeable filter supports. The cells form an epithelial monolayer, with tight junctions separating an apical surface from a basolateral membrane facing the filter support and neighbouring cells. Both surfaces have a unique composition of proteins and lipids [1,2]. Newly synthesized secretory proteins are sorted in the trans-Golgi network and from there transported to the apical and basolateral surfaces. Sorting of proteins to the basolateral surface often relies on proteinaceous signals in cytoplasmically exposed domains of the protein. Association with lipid rafts and glycosylation have been proposed to be involved in apical targeting [3].

As marker proteins of the apical and basolateral plasma membrane of MDCK cells we have previously raised monoclonal antibodies recognizing two membrane glycoproteins. The apical marker protein gp114 is a highly glycosylated integral membrane protein with an apparent molecular weight of 114 kDa [4,5]. The basolateral marker protein has been termed p58 according to apparent molecular weight. In subconfluent monolayers of MDCK cells, p58 localizes to both the basolateral and the apical surface, but later disappears from the apical surface, concomitantly with the development of a tight monolayer [4].

The proteomic identification of membrane proteins of MDCK cells, especially when highly glycosylated, still presents a considerable challenge. First, it is rather difficult to isolate these proteins in sufficient amounts. Second, the canine genome is only partially available and EST sequences do not adequately cover its proteome. Conventional methods of database searching rely heavily on matching masses of intact peptides (peptide mass mapping) or their fragments (tandem mass spectrometry) to the corresponding masses obtained by in silico processing of protein sequences from database entries [6]. Stringent matching of computed and measured masses increases the specificity and the speed of database searching considerably, yet restricts the reach of proteomics methodologies down to a handful of favourably covered model species [7]. Recently developed methods of mass spectrometry driven sequence similarity searches [8,9] utilize redundant, degenerate and partially inaccurate peptide sequences, produced by de novo interpretation of tandem mass spectra and are capable of identifying distant homologues of known proteins from phylogenetically distant organisms [10].

In this work we applied immunoaffinity chromatography to enrich the heavily glycosylated membrane proteins gp114 and p58 and identify them by tandem mass spectrometry and homology driven proteomics.

## Results and discussion

### Enrichment of gp114 by immunoaffinity chromatography

**Figure 1:**
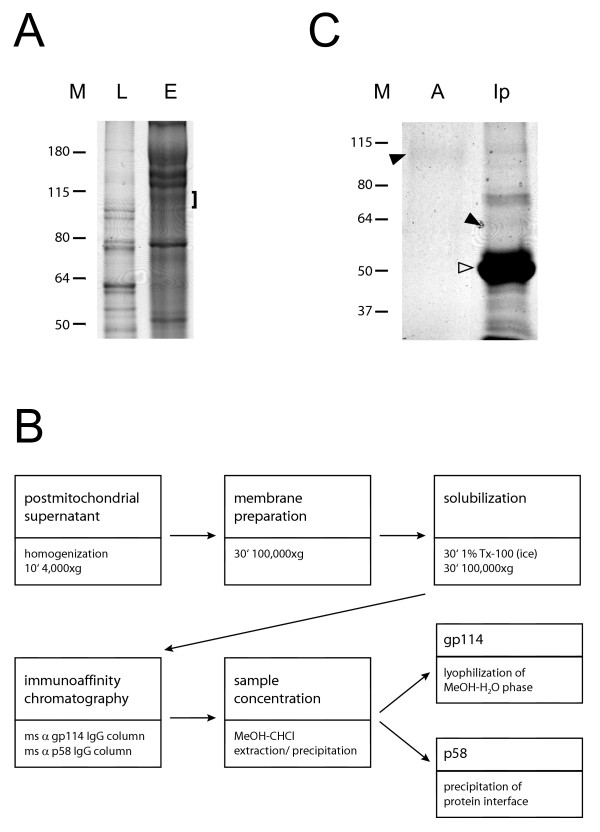
A. WGA lectin affinity chromatography of MDCK cell membrane proteins. Bound proteins were eluted with 0.3 M N-acetylglucosamine, and stained by Coomassie blue after gel electrophoresis. L: aliquot of loaded protein preparation. E: eluted protein pattern (bracket indicates 114 kDa region). B. Flow chart for the purification of gp114. MDCK cell membranes were recovered by high-speed centrifugation from a postmitochondrial supernatant and partially solubilized by treatment with the non-ionic detergent Triton X-100 on ice. Soluble proteins were applied to immunoaffinity columns, and the eluted fractions concentrated by methanol-chloroform extraction-precipitation. Gp114 did not accumulate at the "protein" interface between aqueous and lipid phase but stayed in the hydrophilic supernatant. C. Enrichment of gp114. Lane 1 (A) corresponds to the methanolic phase after chloroform-methanol extraction of eluted proteins from the gp114 immunoaffinity column. Gp114 (arrowhead) is only weakly stained by Coomassie solution. Lane 2 (Ip) contains deglycosylated gp114 (arrowhead) after immunoprecipitation which was confirmed by Western blotting (not shown). The heavy chain of gp114 IgG is indicated by a white arrowhead.

Our first approach was based on the glycoprotein properties of gp114 [5]. A membrane fraction of MDCK cells was enriched for glycoproteins by lectin affinity chromatography using wheat germ agglutinin. The 114 kDa region of the gel electrophoresis pattern (Figure 1a) was analyzed by mass spectrometry. Six peptides matched canine intercellular adhesion molecule 1 (ICAM-1). Other proteins identified were not apical proteins (α2-, β1-integrins, CD44, LAMP-2). A few peptides from low abundant spectra could not be assigned to any protein. Antibodies against dog ICAM-1 immunoprecipitated a 114 kDa protein, but this protein was not recognized by antibodies against gp114 (not shown). Therefore we concluded that gp114 is a protein different from ICAM-1.

Immunoaffinity columns established with mouse anti gp114 IgG were used for a more efficient enrichment of gp114. Detergent soluble membrane protein fractions were applied to immunoaffinity columns, and the eluted fractions concentrated by methanol-chloroform extraction-precipitation. Surprisingly, gp114 did not partition with other proteins, but remained in the aqueous phase, probably due to its high glycosylation (see the flow chart of the purification of gp114, Figure 1b). Gel electrophoresis followed by Coomassie blue staining revealed a single faint band corresponding to gp114 (Figure 1c). In parallel, gp114 was first immunoprecipitated and then enzymatically deglycosylated since we anticipated that the high amount of glycosylation might affect the efficiency of tryptic digestion prior to the analysis by mass spectrometry (Figure 1c).

### Identification of gp114 by mass spectrometry

**Figure 2:**
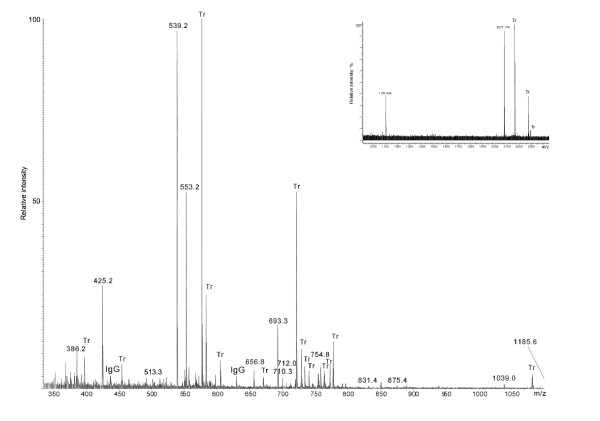
Nanoelectrospray spectrum of in-gel tryptic digest of gp114. Peaks labeled with Tr originated from the autolysis of trypsin; peaks labeled with IgG were identified as tryptic peptides from IgG antibodies by Mascot searches. Tandem mass spectra of other peaks were not matched by Mascot and were subjected to de novo interpretation (Figure 3). Inset: MALDI TOF peptide mass map of the tryptic digest of the gp114 band obtained from the gel shown in figure 1c, lane A. Deglycosylated gp114 gave the same spectrum (not shown). Peaks of autolysis products of trypsin labeled with Tr. Peaks of peptides originating from gp114 are designated with their m/z.

**Figure 3:**
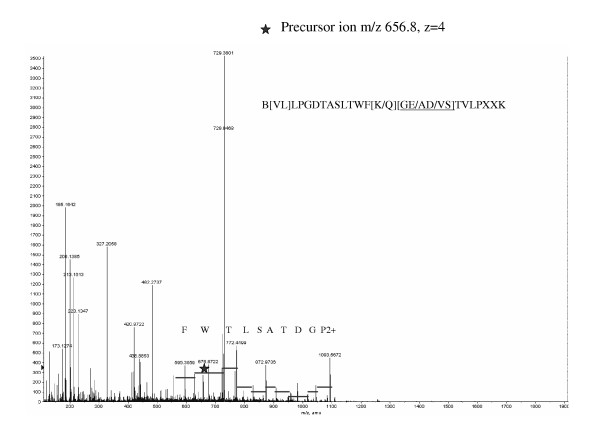
Tandem mass spectrum of a precursor ion with m/z 656.8 and charge +4 (the charge was determined from mass difference between its isotopic peaks). The precursor ion is labeled with an asterisk. The spectrum was partially interpreted by considering precise mass differences between the adjacent fragment ions. Doubly charged fragment series rendered the sequence PGDTASLTWF which was further extended toward the N-terminus using very low abundant ions in the m/z range > 1100 (not shown), but the sequence of the two N-terminal amino acid residues remained ambiguous (VL or LV). It was possible to determine the C-terminal amino acid (K) and a short sequence stretch (TVLP) spaced from the C-terminal lysine by two or three unknown amino acid residues (X). Bridging between the sequence stretches PGDTASLTWF and TVLP could have been achieved by one of the three isobaric combination of amino acid residues, and the order of amino acid residues remained unknown. Hence the peptide might contain the sequence WF-QGET-VL, or WF-QEGT-VL, or WF-QADT-VL, or WF-QDAT-VL..., etc.

The MALDI TOF spectrum of a tryptic digest of the gp114 band contained only two peptide signals, which is a surprisingly low number for a protein of this size (Figure 2, inset). Since MALDI and electrospray spectra acquired from the same digest usually demonstrate different peptide profiles [11], the digest was further investigated by nanoelectrospray tandem mass spectrometry (Figure 2). The Mascot database search with uninterpreted tandem mass spectra gave only three matches to immunoglobulin peptides, although 40 precursor ions were fragmented. The major peptide peaks in the spectrum remained unassigned. Therefore the unmatched tandem mass spectra were manually interpreted de novo by considering mass differences between adjacent peaks of fragment ions (Figure 3). This approach only rendered low confidence amino acid sequences since it is not known if the considered ions indeed belong to the same fragment series [12]. Furthermore, spectra from multiply charged precursor ions contained non-overlapping fragment series with different charge states, which did not cover the complete peptide sequence. Therefore the interpretation of each spectrum produced several inaccurate, partially redundant and incomplete peptide sequence proposals (Table 1). We then merged peptide sequence candidates obtained by the interpretation of all good quality tandem mass spectra into a single search string and employed the mass spectrometry-driven BLAST (MS BLAST) protocol for the identification of proteins by sequence similarity searching [9,10]. The database search confidently hit proteins of the carcinoembryonic antigen (CEA) protein family (Table 1). The table shows only the first protein homologue of the database search, carcinoembryonic antigen-related cell adhesion molecule 8 (CEACAM8), but other CEA family proteins gave the same alignment with an identical score. Remarkably, not a single alignment covered the corresponding sequence completely. The conservation of gp114 across species is apparently not sufficient for an identification by cross-species matching of acquired tandem mass spectra matching using Mascot software [13].

**Table 1:** Peptide sequences of gp114 derived from MS/MS spectra. Peptide sequences from gp114 produced by the interpretation of MS/MS spectra and MS BLAST alignments with corresponding peptides. X are unidentified amino acid residues, L stands for both Leu and Ile residues; B stands for a generic trypsin cleavage site (Arg or Lys); sequences in brackets present isobaric combinations of amino acid residues, which could not be distinguished because of the absence of the corresponding fragment ions in the mass spectrum. All peptide sequence candidates from all fragmented precursors were merged into a single MS BLAST search string. Multiple sequence candidates per each fragmented precursor were allowed. Peptides 2, 4, 5, 3 and 6 (underlined) are contained in one putative exon derived from sequence FE8.

**#**	**m/z**	**z**	**m**	**Sequence proposal**	**Alignment**	**Protein homologue**
1	1185.6	3	3553.8	SNVLYGPDTPTLSQSLGNS	Query: NVLYGPDTPTLSQS	CEACAM8
					NVLYGPD PT+S S	P31997
					Sbjct: NVLYGPDAPTISPS	H. sapiens
						
2	693.3	3	2076.9	BXXTVESVPPNAAEGKDALLR	Query: TVESVPPNAAEGKDALL	CEACAM8
	1039	2			T+E VP NAAEGK+ LL	P31997
					Sbjct: TIEAVPSNAAEGKEVLL	H. sapiens
						
3	553.2	2	1104.4	BVTTPGPAYSGR	Query: TPGPAYSGR	CEACAM8
					TPGPAYS R	P31997
					Sbjct: TPGPAYSNR	H. sapiens
						
4	656.8	4	2623.2	B [VL]LPGDTASLTWFK [W/GE/AD/SV]TVLPXXK	Query: WFKGETV	CEACAM8
	875.4	3			W+KGETV	P31997
					Sbjct: WYKGETV	H. sapiens
						
5	539.2	2	1076.4	BLLLYVLDTK	Query: BLLLYVLDT	Q9NOP7
					+++ YV+ T	CEA protein
					Sbjct: RIIGYVIAT	P. hamadryas
						
6	425.2	2	848.4	BFETALLR	No match	
						
7	386.2	2	770.4	BTVPDSPR	No match	

FE8 putative exon

SLLAFWNPPTTAQVTVESVPPNAAEGKDALLRVLNLPGDTASLTWFKGETVLPTHKILLYVIDTKITTPGPAYSGRETIY PNGSLLFQNITLNDTGSYILQIINQKFETALVRGQLQVFRE

### In silico analysis of gp114

The CEA protein family consists of two separate branches, the membrane associated CEACAM proteins and the soluble pregnancy-specific glycoproteins (PSG). The CEACAM proteins are extensively spliced yielding numerous isoforms. In addition, some CEACAM proteins are modified to include a glycophosphatidylinositol (GPI) anchor instead of a transmembrane domain (reviewed in [14,15]). Gp114 is an integral membrane protein ([16] and references therein) and belongs therefore to the CEACAM subgroup.

MS BLAST searches could only be performed against a protein database. Once gp114 had been identified as a canine CEACAM protein, we used the human CEACAM1 nucleotide sequence to search for homologous genes. One genomic sequence (FE8, see Methods for details) contained an exon sequence homologous to human CEACAM1. Five of the sequenced peptides could be matched exactly to this translated exon sequence (Table 1). Other canine genomic sequences homologous to human CEACAM1 were either identical to FE8 or did not match the sequenced peptides. Thus identical peptides identified in the dog genome validated the sequence similarity identification by MS BLAST. One of the peptides (#6) had also been detected in our first analysis of lectin bound 114 kDa proteins, but could not be assigned at that time. This confirmed that gp114 was indeed present in the lectin bound 114 kDa fraction, but could not be identified on the basis of a single peptide sequence.

While this manuscript was under evaluation, the canine genome became publicly available [17]. A tentative amino acid sequence was obtained (see Methods for details) which was significantly similar to human CEACAM family proteins 1, 5, 8 and 6. CEACAM 5, 8 and 6 are GPI anchored proteins which have been reported to be expressed in humans only [14]. Furthermore, the predicted molecular weights of the mature proteins (without N-glycans) are 54 kDa for human CEACAM1, 71 kDa for CEACAM 5 and 32 kDa for CEACAM 8 and 6. Only the molecular weight of human CEACAM1 corresponds reasonably to the size of deglycosylated gp114 [18]. Deglycosylated gp114 (Figure 1c) gave the same two characteristic fragments as the untreated protein by MALDI TOF analysis (not shown). Other names for CEACAM1 are biliary glycoprotein, BGP1, TM-CEA and CD66a [19]. In summary, gp114 is a dog CEACAM protein, most likely CEACAM1.

### Properties of canine CEACAM/gp114

Apical sorting of gp114/canine CEACAM occurs directly to the surface with a half time of 45 minutes [16]. The glycans are of the N-glycosylated complex type containing sialic acid, contributing about half of the apparent molecular weight of gp114 [5,18]. However, in MDCK-RCA cells deficient in terminal glycosylation due to an inactivated UDP-galactose transporter [20,21], gp114 was missorted to the basolateral surface, whereas targeting of other apical proteins was not affected. Furthermore, endocytosis of gp114 is also highly increased in these cells compared to a very slow internalization in MDCK wild type cells [18]. Independently, gp114 was identified as a major protein undergoing bidirectional transcytosis in MDCK-RCA cells [22]. Antibody crosslinking shows that gp114 coclusters with lipid raft associated proteins in the apical membrane of MDCK cells [23].

Lipid raft microdomain association and glycosylation dependent trafficking (basolateral missorting, endocytosis, transcytosis) have not been reported for CEACAM proteins so far. Reversible association with lipid microdomains has been put forward as a core mechanism in the regulation of signal transduction at the plasma membrane [24]. Our identification enables the integration of the data obtained for gp114 with the characterization of CEACAM proteins from other approaches.

### Identification of p58

The p58 protein was enriched by immunoaffinity chromatography, similarly to gp114. The trypsin digested band of p58 was identified as the β-chain of canine Na^+^K^+^-ATPase by peptide mass fingerprinting. 15 peptides were matched to the masses of corresponding tryptic peptides with better than 100 ppm mass tolerance. The MOWSE score of 143 exceeded the significance threshold of 72 and thus the identification was considered confident.

The β-subunit of Na^+^K^+^-ATPase contains three N-linked glycans, which is consistent with the apparent molecular weight of the expressed protein. The association of the β-subunit with the α-subunit is required for the enzyme complex to reach the plasma membrane (for a review, see [25]). The polarized expression of Na^+^K^+^-ATPase in epithelia depends on the association of β-subunits from neighbouring cells [26]. The molecular weight of the α-subunit corresponds to the protein coprecipitating with p58 antibodies under non-denaturing conditions (not shown). Na^+^K^+^-ATPase has also been used in other cell systems as a basolateral marker protein.

### MS identification of proteins from organisms with unsequenced genomes

Mass spectrometry driven sequence similarity searches now make it possible to characterize proteins from model organisms with unsequenced genomes by their similarity to already available sequences. Computational simulations suggested that almost all proteins within mammalian phylogenetic lineage could be identified by MS BLAST sequence similarity searches using 10 sequenced tryptic peptides, which is a rather frequent outcome of tandem mass spectrometric experiments [10]. Importantly, the method imposes rather loose requirements on the quality of peptide sequences and thus paves the way to complete automation of the analytical routine. Mass spectrometric characterization of unknown proteins can be performed in a layered approach [7,27] i.e. conventional proteomics methods could be applied first to identify highly conserved proteins that share identical peptide sequences with their known homologues, and sequence similarity searches would only be applied to a selection of non-conserved proteins once the conventional methods failed. Thus we might anticipate that the scope of proteomics methods will be able to support biochemical research in any vertebrate model.

## Conclusion

The apical marker glycoprotein gp114 has been enriched from tissue culture cells and identified by tandem mass spectrometry as canine carcinoembryonic antigen-related cell adhesion molecule (CEACAM). We exemplify the difficulties associated with identifying glycoproteins from model systems without sequenced genomes, and how to overcome them. The general strategy provides a framework which should be useful for many related approaches.

Known properties of gp114 such as glycosylation dependent transcytosis and association with lipid microdomains involved in signal transduction can now be integrated with the knowledge about CEACAM proteins obtained by different approaches.

## Methods

### IgG-protein G sepharose columns

A membrane fraction from dog intestine was used for generation of monoclonal antibodies 4.6.5a (gp114) and 6.23.3 (p58) [4]. Hybridoma cells 4.6.5a and 6.23.3 were grown in serum-free HyQ SFX-MAb medium (HyClone, Logan, Utah) for two weeks. Supernatants were clarified by sequential centrifugation at 200 × g and 10,000 × g, adjusted to 20 mM HEPES pH 7.2 and filter sterilized. Supernatant containing 1 mg of IgG was crosslinked with dimethylpimelimidate to immobilized protein G according to supplier's recommendations (Pierce, Rockford IL).

### Membrane preparation

MDCK cells were grown on plastic dishes corresponding to a surface area of 0.9 m^2 ^equivalent to 3.6 × 10^9 ^cells. A postmitochondrial supernatant was obtained by homogenizing cells in 0.25 M sucrose, 3 mM imidazol pH 7.4 (13× pushing through a 22-gauge needle) and centrifugation at 4,000 × g. Membranes were pelleted for 30 minutes at 100,000 × g, and treated on ice for 30 minutes with TNE1 (20 mM Tris pH 7.4, 150 mM NaCl, 5 mM EGTA) containing 1% w/v Triton x-100. Under these conditions, p58 and gp114 are efficiently solubilized. Not solubilized membranes were removed by centrifugation at 100,000 × g for 30 minutes, and the supernatant (19 mg total protein) used for immunoaffinity chromatography.

### WGA lectin affinity chromatography

Solubilized membranes were adjusted to WGA buffer (10 mM HEPES 7.4, 1 mM MgCl_2_, 1 mM CaCl_2_, 150 mM NaCl, 0.1% Tx-100) and circulated o/n over a 5 ml WGA-agarose (AmershamPharmacia) column at 0.2 ml/min. Washing was with 10× column volume of WGA buffer, and elution in 1 ml steps with WGA buffer containing 0.3 M N-acetylglucosamine.

### Immunoaffinity chromatography

The solubilized membrane preparations were passed three times over the IgG-protein G sepharose columns. Columns were washed with 50 ml of TNE2 (10 mM Tris pH 7.4, 150 mM NaCl, 1 mM EDTA) containing 0.1% w/v Tx-100. Elution with 0.1 M glycine pH 2.6, 0.1% w/v Tx-100 was in 1 ml steps. Eluted fractions were neutralized, concentrated by spin columns (Centrikon YM-30), and then desalted and precipitated by methanol/chloroform extraction [28]. Aqueous supernatants were lyophilized and found to contain high amounts of gp114, but no p58. Highly glycosylated proteins have been reported to partition into the aqueous phase under these conditions [29].

Immunoprecipitation followed by enzymatic deglycosylation with PNGaseF (Roche) was according to standard procedures.

### Mass spectrometry

Proteins separated by polyacrylamide gel electrophoresis were visualized by Coomassie staining, and bands were excised and digested by trypsin (Promega) as described [30]. 1 μl aliquots of digests were analyzed by MALDI peptide mapping on a Reflex IV MALDI TOF mass spectrometer (Bruker Daltonics, Germany) using AnchorChip™ targets as described [31]. Tryptic peptides were extracted from the gel matrix by 5% formic acid and acetonitrile, pooled and lyophilized. Peptides were sequenced by nanoelectrospray tandem mass spectrometry on a QSTAR Pulsar i quadrupole time-of-flight mass spectrometer (MDS Sciex, Canada). 40 tandem mass spectra were acquired from the digest of the gp114 band. Uninterpreted tandem mass spectra were first used to search a protein sequence database MSDB using Mascot software (Matrix Science Ltd, UK) v.1.8 installed on a local server. No restrictions on species of origin or protein molecular weight were imposed. All Mascot hits were further verified by manual inspection of matched tandem mass spectra. Spectra, which were not matched by Mascot, were manually interpreted de novo. The interpretation of each spectra rendered a few degenerate, redundant and incomplete peptide sequence candidates, which were assembled into a single MS BLAST [9] query string as described previously [32]. MS BLAST searches against the non-redundant protein database nrdb95 were performed on a web server [33].

### In silico analysis

The human CD66a sequence was blasted against a dog genomic database [34]. The best match was obtained with sequence G630P617675FE8.T0, which was then translated into the amino acid sequence. After the dog genome became available, we repeated our homology searches. Since the FE8 data showed that four of the sequenced peptides (#2-#4-#5-#3) form an almost continuous stretch, we could probe the new (nucleotide) databases now with a 65 amino acid sequence. Eight significant alignments were found, all on chromosome I. The top three alignments were investigated more closely (exclusion limit: better than 90% over 60 amino acids, taking into account that MS cannot distinguish between Ile and Leu or isobaric amino acid combinations). Only one translated nucleotide sequence contained peptides #1 and #6. Using human CEACAM family proteins for guidance, a tentative amino acid surfaced out of merging putative exons. This sequence was 58–61% identical and 67–71% similar to human CEACAM family proteins 1, 5, 8 and 6.

## Abbreviations

CEA, carcinoembryonic antigen

CEACAM, carcinoembryonic antigen-related cell adhesion molecule

MDCK, Madin-Darby canine kidney

WGA, wheat germ agglutinin

## Authors' contributions

J. F. initiated and designed the study, did all biochemical work and wrote the manuscript. Anna S. did the mass spectrometry analysis, Andrej S. and K. S. co-wrote the manuscript.
